# Identification of benzazole compounds that induce HIV-1 transcription

**DOI:** 10.1371/journal.pone.0179100

**Published:** 2017-06-28

**Authors:** Jason D. Graci, Daniel Michaels, Guangming Chen, Gillian M. Schiralli Lester, Sarah Nodder, Marla Weetall, Gary M. Karp, Zhengxian Gu, Joseph M. Colacino, Andrew J. Henderson

**Affiliations:** 1 PTC Therapeutics, Inc., South Plainfield, New Jersey, United States of America; 2 Department of Medicine and Microbiology, Section of Infectious Diseases, Boston University School of Medicine, Boston, Massachusetts, United States of America; 3 Department of Pediatrics, Neonatology, School of Medicine and Dentistry, University of Rochester Medical Center, Rochester, New York, United States of America; George Mason University, UNITED STATES

## Abstract

Despite advances in antiretroviral therapy, HIV-1 infection remains incurable in patients and continues to present a significant public health burden worldwide. While a number of factors contribute to persistent HIV-1 infection in patients, the presence of a stable, long-lived reservoir of latent provirus represents a significant hurdle in realizing an effective cure. One potential strategy to eliminate HIV-1 reservoirs in patients is reactivation of latent provirus with latency reversing agents in combination with antiretroviral therapy, a strategy termed “shock and kill”. This strategy has shown limited clinical effectiveness thus far, potentially due to limitations of the few therapeutics currently available. We have identified a novel class of benzazole compounds effective at inducing HIV-1 expression in several cellular models. These compounds do not act via histone deacetylase inhibition or T cell activation, and show specificity in activating HIV-1 in vitro. Initial exploration of structure-activity relationships and pharmaceutical properties indicates that these compounds represent a potential scaffold for development of more potent HIV-1 latency reversing agents.

## Introduction

Use of antiretroviral therapy (ART) suppresses HIV-1 replication and decreases the morbidity and mortality of HIV-1-associated diseases. However, even after long-term suppression of viral replication, HIV-1 rapidly rebounds after ART is discontinued [1–6]. Although incomplete inhibition of viral replication may contribute to this phenomenon [7], the rebound is likely due to the early establishment of a stable reservoir of latently infected cells [1–4,8–10]. These cellular reservoirs remain unrecognized by the host immune response and contribute to increased viremia upon ART interruption [3,4,11]. Importantly, long-lived quiescent CD4+T cell subsets, including T memory cells, are key reservoirs of latent infection and it has been estimated that it would take over 60 years of ART to eliminate this population in an individual [12]. Thus, under current treatment modalities, life-long ART is needed to maintain suppression of HIV-1. Limitations of current ART include the emergence of viral resistance [13], cumulative side effects [14–16], and an unbearable financial burden for regions of the world hit hardest by the epidemic. With nearly 37 million people already infected with HIV-1, and 2 million new infections and 1.2 million HIV/AIDS-related deaths worldwide in 2014 [17], the need for novel therapies that eliminate HIV-1 infection is of high priority.

The mechanisms underlying HIV-1 latency are not fully characterized, and evidence suggests that multiple processes maintain the latent provirus [18–20]. Factors that contribute to proviral latency include: 1) a lack of expression of appropriate transcription factors in resting cells [21,22]; 2) silencing of viral gene expression due to chromosome structure or epigenetic modifications at the site of provirus integration [23–26]; 3) premature transcriptional termination due to insufficient levels of Tat and associated host factors [27–29]; 4) ineffective transport of viral RNAs encoding the late viral proteins, such as Gag, Pol and Env (for review see [19]); 5) transcriptional interference [30]; and 6) silencing of viral gene expression via microRNAs [31,32]. It is still not clear which of these mechanisms are responsible for the establishment and maintenance of latency in HIV-infected individuals.

A number of strategies have been proposed to eliminate the reservoir of latent HIV-1in infected patients, including those employing CRISPR/Cas9 or zinc-finger nuclease gene editing, chimeric antigen receptor (CAR) modified T cells, and therapeutic vaccination to elicit broadly neutralizing antibodies (reviewed in [33]). Another proposal to effect a cure for HIV-1 infection has been termed “shock and kill” [34]. In this approach, patients remain on ART to prevent new infections while undergoing simultaneous treatment with a latency reversing agent, a therapeutic regimen capable of stimulating HIV gene expression in latently-infected cells. The cells from which the latent proviruses are activated are anticipated to die due to apoptotic effects of viral expression and/or immune system recognition of cells that have been induced to express viral proteins [1,35]. There have been a number of unsuccessful attempts to flush the latent virus from infected individuals via non-specific activation of resting T cells, such as anti-CD3 or IL-2 treatments [36–39]. Recently, small molecules targeting epigenetic factors have been explored as agents to reactivate latent provirus (reviewed in [40]), including histone deacetylase (HDAC) inhibitors, methyltransferase inhibitors, and protein kinase C activators. Clinical trials with HDAC inhibitors have failed to decrease significantly the size of the latent reservoir in HIV infected patients [41–47], highlighting the need for additional small molecules that target HIV latency.

Here we describe the discovery of a novel class of benzazole compounds that stimulate HIV-1 proviral expression in cellular models, are selective with regards to cytotoxicity and stimulation of other latent viruses, and do not act through T cell stimulation or HDAC inhibition. These compounds represent a potential starting point to discover more potent latency antagonists and as molecular probes to further understand the determinants of HIV-1 latency.

## Materials and methods

### Cells and reagents

Jurkat, SupT1, HeLa, BCP-1, and Raji cell lines were purchased from ATCC (Manassas, VA). 24STNLSG cells were licensed from The University of Medicine and Dentistry of New Jersey, New Brunswick, NJ (UMDNJ, now part of Rutgers University)[48]. ACH-2 and U1 cells were obtained through the NIH AIDS Reagent Program, Division of AIDS, NIAID, NIH. Cryopreserved PBMCs were purchased from AllCells, LLC (Alameda, CA). RPMI-1640, DMEM, hygromycin B, penicillin/streptomycin and fetal bovine serum (FBS) were purchased from Invitrogen/Thermo Fisher Scientific (Waltham, MA). Recombinant tumor necrosis factor (TNF)-α was purchased from R&D Systems (Minneapolis, MN). Ampicillin sodium salt, phorbol 12-myristate 13-acetate (PMA), valproic acid (VA), sodium butyrate (NaBu), and trichostatin A (TSA) were purchased from Sigma-Aldrich (St. Louis, MO). Suberanilohydroxamic acid (SAHA) was purchased from Cayman Chemical (Ann Arbor, MI). JQ1 was obtained from the Bradner laboratory (Harvard Medical School, Boston, MA). Compound **1**, 2-(4-(5-amino-1H-benzimidazol-2-yl)phenyl)benzoxazol-5-amine, was acquired from ChemDiv (San Diego, CA). Compounds **2–6** were synthesized at PTC Therapeutics, Inc.

### SEAP assay in 24STNLSG cells

SupT1-derived 24STNLSG cells [48,49] were plated at 100,000 cells/well in black bottom/black walled 96-well plates in 50 μl RPMI-1640 medium (without phenol red) containing 10% FBS. Compound was added in 50 μl of identical medium using three-fold dose-response dilutions, in duplicate. All wells had a final DMSO concentration of 0.5%. TNF-α, a nuclear factor-κB (NF-κB) activator known to induce HIV- expression in cellular models [50], was used as a positive control. Plates were incubated for 72 h at 37°C, 5% CO_2_. Plates were then sealed and heated to 65°C for 30 min. Plates were brought to room temperature and 50 μl assay buffer (1 mM MgCl_2_, 10 mM L-homoarginine, 1 M diethylamine hydrochloride) was added to each well and incubated for 5 min at room temperature. To each well, 50 μl of CSPD Substrate (0.4 mM Ready-to-Use with Sapphire-II Enhancer; Applied Biosystems, Foster City, CA) was added. Plates were incubated for 20 min at room temperature and read on a ViewLux microplate imager (Perkin Elmer, Waltham, MA). Curves were fit by non-linear regression using Prism software (GraphPad, San Diego, CA).

### Cytotoxicity assays

SupT1 cells were plated at 100,000 per well in 96-well, white-walled plates with serial dilutions of test compound in a total volume of 100 μl RPMI-1640 medium containing 10% FBS and 0.5% DMSO. PBMCs were plated similarly at 50,000 cells/well and stimulated by addition of 40 μg/ml PHA and 80 ng/ml interleukin-2 (IL-2). Cells were incubated for 72 h at 37°C, 5% CO_2_. ATP was quantified using the CellTiter-Glo Luminescent Cell Viability Assay (Promega, Madison, WI) on a ViewLux microplate imager (Perkin Elmer, Waltham, MA). Percent cytotoxicity was determined relative to vehicle-only treated wells. CC_50_ values (50% cytotoxic concentration) were determined by non-linear regression using Prism software (GraphPad, San Diego, CA).

### HIV activation assay in ACH-2 model of latency

ACH-2 cells were maintained in RPMI-1640 medium without phenol red and with 10% FBS at 37°C in a 5% CO_2_ atmosphere. Cells were plated at 5000/well in 96-well round-bottom plated and test compounds were added in a final concentration of 0.5% DMSO, in duplicate. Cells were incubated for 24 h at 37°C, 5% CO_2_, then pelleted at 2000 × g for 5 min followed by washing in 0.1 ml PBS. RNA was isolated using Invitrogen PureLink 96 RNA Purification Kit with vacuum manifold per the manufacturer’s instructions, including DNase I treatment. HIV-1 transcripts were detected using TaqMan^®^ RNA-to-C_T_^™^ 1-step kit (Applied Biosystems, Foster City, CA) and primers/probe specific to *gag* [51] (SK38 forward: 5ʹ-ATA ATC CAC CTA TCC CAG TAG GAG AAA T-3ʹ; SK39 reverse: 5ʹ-TTT GGT CCT TGT CTT ATG TCC AGA ATG C-3ʹ; SK19 probe: 5ʹ-6FAM ATC CTG GGA TTA AAT AAA ATA GTA AGA ATG TAT AGC CCT AC TAMRA-3ʹ) or *tat* [52] (forward: 5ʹ-GCC TTC ATT GCC AAG TTT GTT T-3ʹ; reverse: 5ʹ-GTC GCT GTC TCC GCT TCT TC-3ʹ; probe: 5ʹ-6FAM CAA GAA AAG GCT TAG GCA TCT CCT ATG GCA TAMRA-3ʹ). GAPDH RNA was monitored in all assays as an endogenous control for selectivity using a pre-developed TaqMan assay reagent (Applied Biosystems, Foster City, CA). RNA was quantified on an Applied Biosystems 7900HT. PCR replicates were performed for each biological replicate. Viral RNA expression was normalized to GAPDH RNA and data are expressed as the change from vehicle-only control wells. Curves were fit by non-linear regression using Prism software to determine EC_10×_ (concentration of compound resulting in 10-fold increase in viral RNA).

### Intracellular staining for HIV Gag

ACH-2 cells were treated as indicated and HIV-1 expression was monitored by intracellular staining for p24. Approximately, 1 × 10^6^ cells were collected and washed in PBS before fixing by resuspending in 100 μl of BD Cytofix/Cytoperm (BD Biosciences, San Jose, CA) at 4°C for at least 20 min. Cells were then harvested by centrifugation and resuspended in 100 μl BD PermWash (50 mM NH_4_Cl, 5 mM EDTA, 0.1% saponin, 0.5% BSA, PBS and 1 mg/ml of anti-HIV-Gag PE (Clone KC57, Beckman Coulter, Brea CA). Cells were stained for 30 min, washed with the PermWash, and resuspended in FACS buffer (2 mM EDTA, 0.5% BSA, 1% paraformaldehyde in PBS). Cells were analyzed by flow cytometry using a FACSCalibur instrument (BD Biosciences). Flow cytometry was performed at the Boston University School of Medicine flow cytometry core.

### IL-2 ELISA

Cryopreserved PBMCs from a single donor were thawed in RPMI-1640 plus 10% FBS. PBMCs were plated at 5×10^4^/well in 96-well plates and compound was added with a final concentration of 0.5% DMSO. Plates were incubated for 24 h at 37°C, 5% CO_2_. Interleukin-2 was quantified using Human IL-2 DuoSet ELISA (R&D Systems, Minneapolis, MN). Raw data were converted to ng/ml IL-2 using a standard curve obtained in parallel.

### Transfection and reporter assays

The vector pGL4.32[*luc2P*/NF-κB-RE/Hygro] was purchased from Promega (Madison, WI), encoding 5 copies of a NF-κB response element driving expression of synthetic firefly luciferase gene, luc2, including a PEST destabilization sequence. Jurkat cells were transfected with this vector using a Gene Pulser^®^ II (Biorad, Hercules, CA) with a 0.4 cm gap cuvette at 0.2 kV and 960 μF. Cells were allowed to recover for three days in RPMI-1640 with 10% FBS and 1% penicillin/streptomycin before selection with 200 μg/ml Hygromycin B for one month. Pooled, stably-transfected cells were plated at 50,000/well in 96-well plates and treated with test compounds in a final concentration of 0.5% DMSO for 4 h at 37°C. Luciferase activity was determined by adding one volume 2× steadylite plus reagent (Perkin Elmer, Waltham, MA) and reading on a ViewLux microplate imager.

The vector pGL4.77[hRlucP/Hygro] encoding a Renilla luciferase gene including a PEST destabilization sequence, was purchased from Promega (Madison, WI). HIV-1 cDNA was generated from HIV_IIIB_ virus using PureLink^®^ Viral RNA/DNA Mini Kit (Invitrogen). The HIV-1 core promoter region (nucleotides -105 to 60) was amplified from HIV_IIIB_ cDNA using primers designed with NheI (forward: 5ʹ-GCT AGC GGG ACT TTC CGC TGG G-3ʹ) and XhoI (reverse: 5ʹ-CTC GAG AGT GGG TTC CCT AGT TAG CCA-3ʹ) flanking restriction site sequences. DNA fragments were generated using Platinum^®^ Taq DNA Polymerase High Fidelity (Invitrogen) and fragment size was verified by electrophoresis using E-Gel^®^ 1.2% agarose (Invitrogen). Fragments were cloned into pCR^™^2.1 TOPO^®^ (Invitrogen), transformed into One Shot^®^ TOP10 Chemically Competent *E*. *coli* (Invitrogen), and selected on LB-agar plates containing 100 μg/ml ampicillin. Individual colonies were grown in LB broth, DNA was isolated using QIAprep^®^ Spin Miniprep Kit (Qiagen), and the inserted fragment was verified by EcoRI digestion followed by electrophoresis using E-Gel^®^ 1.2% agarose (Invitrogen). To insert the HIV-1 LTR into the vector multiple cloning region, the resultant plasmids and pGL4.77[hRlucP/Hygro] were dually digested with NheI and XhoI (New England Biolabs), ligated using ExpressLink^™^ T4 DNA Ligase (Invitrogen), transformed into One Shot^®^ TOP10 Chemically Competent *E*. *coli* (Invitrogen), and selected on LB-agar plates containing 100 μg/ml ampicillin. Clones were picked and DNA isolated using QIAprep^®^ Spin Miniprep Kit (Qiagen) and the inserted fragment was verified by NheI/XhoI digestion followed by electrophoresis using E-Gel^®^ 1.2% agarose (Invitrogen). The resultant plasmid, pGL4.77-LTR, was verified by sequencing at GENEWIZ (South Plainfield, NJ). To assay for LTR promoter activity, 10,000 HeLa cells were plated per well of a 96-well plate in DMEM with 10% FBS and were transfected with pGL4.77-LTR using FuGENE^®^ 6 Transfection Reagent (Promega, Madison, WI) according to the manufacturer’s protocol. One hour post transfection, compounds were added in a final concentration of 0.5% DMSO and plates were incubated at 37°C for 24–48 h. Luciferase activity was quantified using Renilla Luciferase Assay System (Promega, Madison, WI) and results were normalized to the vehicle-treated control.

### KSHV and EBV activation assays

Raji cells, which harbor the Epstein-Barr virus (EBV) genome, or BCP-1 cells, which contain the Kaposi's sarcoma associated herpesvirus (KSHV) genome, were plated at 5000 or 50,0000 cells/well, respectively, in 96-well plates in 50 μl RPMI-1640 with 10% FBS. Compound was added in 50 μl of identical medium using three-fold dose-response dilutions. All wells had a final DMSO concentration of 0.5%. PMA was employed as a positive control. Plates were incubated for 24 h at 37°C, 5% CO_2_. After incubation, RNA was isolated using PureLink^™^ 96 Total RNA Purification Kit. Viral transcripts were detected using TaqMan^®^ RNA-to-C_T_^™^ 1-step kit. For KSHV, ORF50 transcript was detected using primers 5ʹ-CAC AAA AAT GGC GCA AGA TGA-3ʹ and 5ʹ-TGG TAG AGT TGG GCC TTC AGT T-3ʹ and probe 5ʹ-6FAM-AGA AGC TTC GGC GGT CCT G -TAMRA-3ʹ [53]. For EBV, BZLF1 transcript was detected using primers 5ʹ-ACG CAC ACG GAA ACC ACA A-3ʹ and 5ʹ-CTT AAA CTT GGC CCG GCA TT-3ʹ and probe 5ʹ-6FAM-AAT CGC ATT CCT CCA GCG ATT CTG G-TAMRA-3ʹ [54]. GAPDH RNA was monitored in all assays as an endogenous control for selectivity using a pre-developed TaqMan assay reagent (Applied Biosystems, Foster City, CA). RNA was quantified on an Applied Biosystems 7900HT. Viral RNA expression was normalized to GAPDH RNA and data are expressed as change from vehicle-treated control wells.

### Histone deacetylase (HDAC) inhibition assays

HDAC inhibition was determined using HDAC Fluorometric Activity Assay/Drug Discovery Kit (BIOMOL International, Inc.) according to the manufacturer’s recommendations. HeLa cell nuclear extracts and purified HDACs 1, 2, 3, and 8 were obtained from BIOMOL. Briefly, HDAC inhibitors and test compounds were diluted to 5× concentration in assay buffer in a white, flat-bottom 96-well plate. HDAC samples (purified HDAC or HeLa nuclear extract) were diluted to the desired concentration in assay buffer and added to each well. HeLa extract was employed at a 150-fold dilution, while individual HDACs were tested at 0.3–9 μg/ml. Fluor de Lys^™^ substrate was diluted to 2× concentration in assay buffer and the HDAC reaction was initiated by addition of substrate to each well followed by incubation for 60 min at room temperature. A final concentration of 0.4% DMSO and 50 μM Fluor de Lys^™^ substrate was present in all samples. Samples with no enzyme were used as blanks and vehicle-only samples were used as negative controls. HDAC reactions were quenched by adding Fluor de Lys^™^ developer containing 2 μM trichostatin A. Plates were incubated at room temperature for 15 min prior to reading fluorescence for 0.1 s with an excitation wavelength of 360 nm and emission of 460 nm using a 5 nm slit on a Cary Eclipse Fluorescence Spectrophotometer (Varian, Inc.). All samples were blank-subtracted and normalized to HDAC activity of the negative control (set to 100%).

### Caco-2 assay

Caco-2 cells obtained from ATCC were plated on filters of a 24-well Boyden chamber and allowed to replicate for five days. Prior to initiating a study, the integrity of the cell layer was assessed by measuring the transepithelial electrical resistance (TEER) values. To perform the assay, the medium was removed, and the test compound (10 μM) or control (caffeine or atenolol) was added to the top chamber. After 1h, medium was removed from the bottom chamber. After completion of the assay, the integrity of the cell layer was measured by transfer of the fluorescent dye, Lucifer yellow, across the cell layer. Samples taken from the apical chamber at the start of the study and from the basal chamber at the end of the study were assessed for drug levels using LC-MS/MS technology and used to calculate the apparent permeability (P_app_).

### Microsome assays

Human and mouse liver microsomes were obtained from XenoTech, LLC (Kansas City, KS). The reaction mixture was composed of liver microsomes (0.5 mg/mL), 20 mM Tris-HCl pH 7.4, 2.5 mM MgCl_2_, and an NADPH generating system (0.1 mM NADP, 1.2 mM glucose-6-phosphate and 0.3 units/ml glucose-6-phophate dehydrogenase). Verapamil and disopyramide, which are highly and modestly metabolized, respectively, were used as comparator controls. Test compounds were added to the microsome reaction and loss of parent molecule was monitored after 1 h by LC/MS/MS.

### Protein binding

Binding of test compound to plasma proteins was estimated by incubation of test compounds with mouse or human plasma. Human plasma was obtained from BioreclamationIVT (Westbury, NY). Test compound (10 μM) or reference compounds, (*RS*)-warfarin and propranolol, were separately incubated in mouse or human plasma (pH 7.4). After a 15 min incubation at 37°C, triplicate aliquots of the incubation mixtures were transferred to a Millipore Multiscreen Ultracel-10 filter plate (10 kDa molecular mass cut off, EMD Millipore, Billerica, MA). The plate was centrifuged for 60 min at 3000 × g and 37°C. After centrifugation, aliquots of both the ultrafiltrates as well as the incubation mixtures prior to ultrafiltration were analyzed by LC-MS/MS to quantify the concentrations of test compound and reference compounds.

### Pharmacokinetics

All animal studies were performed in compliance with IACUC approved protocols at AAALAC-certified animal facilities. Pharmacokinetics of test compounds were evaluated in CD-1 mice (Charles River Laboratories, Wilmington, MA). Compounds were formulated as a suspension in 0.5% hydroxypropylmethyl cellulose with 0.1% Tween 80 and administered by gavage at a dose of 10 mg/kg. No mice showed evidence of distress or pain after dosing. Mice were anaesthetized with CO2 followed by terminal exsanguination as per the Guide for the Care and Use of Laboratory Animals. Blood was collected by terminal cardiac puncture at 1, 2, 4, 7, 16, and 24 h post-dose (3 mice per time point), and centrifuged to collect plasma. Brain tissue was collected at the time of blood collection and homogenized in water. The concentrations of test compound in plasma and brain tissue were quantified by liquid chromatography-tandem mass spectrometry (LC-MS/MS). Briefly, the plasma and tissue homogenate samples were treated with an acetonitrile-methanol mixture containing an internal standard that is a close structural analog of the test compounds. The treated plasma and brain homogenate samples were centrifuged and the supernatant was collected and analyzed using electro-spray LC-MS/MS.

### Ethics statement

All animal studies were performed at the Rutgers-Robert Wood Johnson Medical School facility, which is approved by the Association for Assessment and Accreditation of Laboratory Animal Care (AAALAC). All procedures, including maintenance and determination of experimental endpoints, were carried out in strict compliance with the Rutgers Animal Care and Use Committee guidelines and all protocols were approved by the Rutgers Institutional Animal Care and Use Committee (IACUC). Mice were group housed in solid bottom cages (5 mice per cage). Mice had more than 15 in^2^ per animal as per Rutgers IACUC policy with free access to food and water.

### Data analysis

Data were plotted with Prism (Graphpad, San Diego, CA). Data are presented as the mean ± SD or SEM as indicated. A sigmoid dose-response with variable slope regression curve was generated for determination of CC_50_ values.

## Results

### Discovery of a benzazole scaffold of compounds that induces HIV-1 transcription

**Fig 1:**
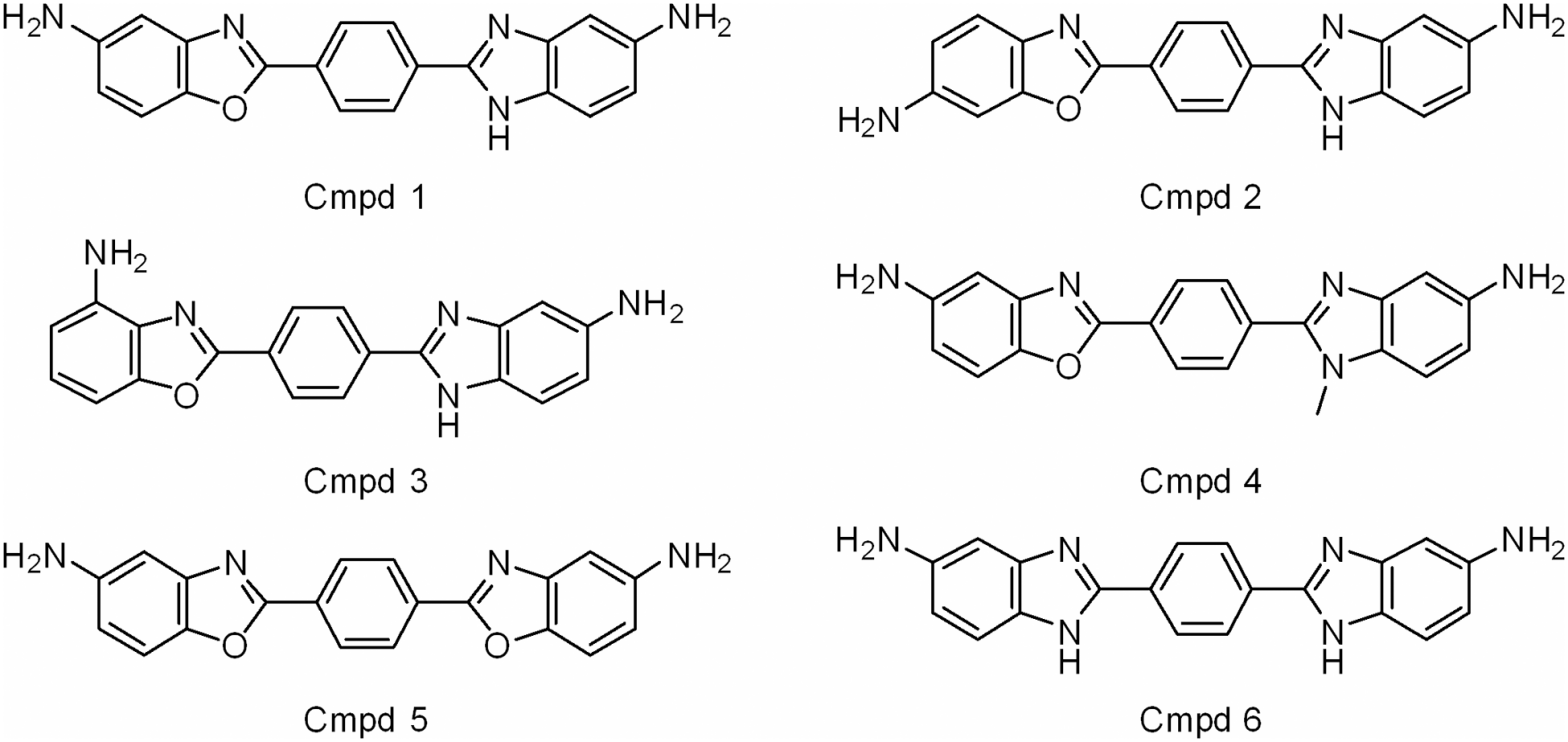
Structures of compounds that induce HIV transcription. Compound **1** was identified through high throughput screening utilizing 24STNLSG cells. A series of analogs (Compounds **2–6**) were synthesized as a preliminary investigation of structure-activity relationship.

**Fig 2:**
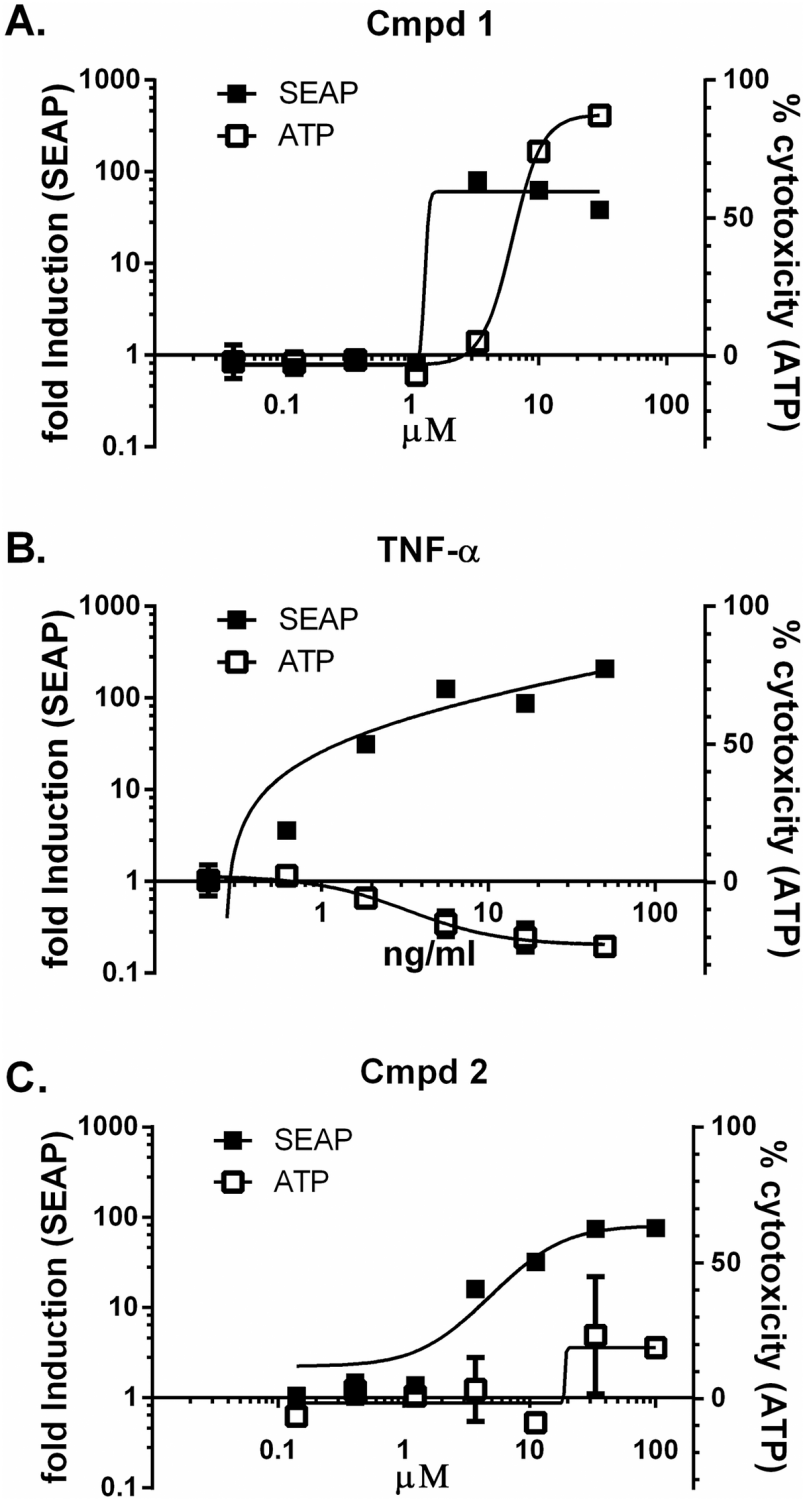
Compounds induce HIV-1 transcription in cellular models. Compound **1** (A), TNF-α (B), and Compound **2** (C) were evaluated in the 24STNLSG cell model by measuring increase in SEAP reporter activity 72 hours post addition (closed symbols, left axis). Cellular ATP was quantified in parallel as a measure of cytotoxicity (open symbols, right axis). Data is presented as mean and standard deviation of 2 replicates per compound concentration. Experiment is representative of at least five independent experiments for each compound.

We identified Compound **1** (Fig 1) via high throughput screening using a previously described latency reactivation assay in 24STNLSG cells [48,49]. Compound **1** increased HIV transcription in this cell line up to 80-fold as measured by a secreted alkaline phosphatase (SEAP) reporter (Fig 2A and Table 1). This compared favorably with TNF-α stimulation of this cell line (Fig 2B). Cytotoxicity was determined in parallel by quantification of levels of cellular ATP.

**Table 1:** Activity and selectivity of compounds that induce HIV-1 transcription.

Compound	EC_2X_, 24STNLSG (μM)	Max. fold induction, 24STNLSG	CC_50_, SupT1 (μM)	EC_10X_, ACH-2 (μM)	CC_50_, PBMC (μM)
**Cmpd 1**^a^	**1.5 ± 0.7**	**80**	**5.2 ± 1.1**	**0.39 ± 0.12**	**18 ± 6.8**
**Cmpd 2**^a^	**2.7 ± 2.3**	**76**	**>100**	**0.97 ± 0.50**	**>100**
**Cmpd 3**^b^	**3.7**	**3**	**8.8**	**N.D.**	**N.D.**
**Cmpd 4**^b^	**>100**	**N.A.**	**>100**	**N.D.**	**N.D.**
**Cmpd 5**^b^	**>100**	**N.A.**	**>100**	**N.D.**	**N.D.**
**Cmpd 6**^b^	**>100**	**N.A.**	**>100**	**N.D.**	**N.D.**

EC_2X_, concentration for 2-fold increase in SEAP activity; CC_50_, 50% cytotoxic concentration; EC_10X_, concentration for 10-fold increase in HIV-1 RNA as determined by qRT-PCR. Values are the mean ± SD. CC_50_ was determined by ATP quantification.

^a^Values derived from 3 independent experiments.

^b^Values are from a single experiment performed in duplicate.

Compound **1** was identified as a benzazole containing 5-aminobenzoxazole and 5-aminobenzimidazole that are connected through a benzene linkage. An analog, Compound **2** (Fig 1), was obtained by moving the amino group on the benzoxazole ring from the 5-position to the 6-position and was found to be less toxic than Compound **1** and activated HIV-1 transcription to similar levels in 24STNLSG cells (Fig 2C and Table 1). Moving the amino group from the 5-position to the 4-position of the benzoxazole led to Compound **3** (Fig 1) which exhibited much weaker activity, causing a maximum of 3-fold induction of HIV transcription in 24STNLSG cells (Table 1). Interestingly, Compound **4** (Fig 1), obtained by a simple methylation of the N1 of the benzimidazole ring, led to a complete loss of activity (Table 1). Either replacing 5-aminobezoxazole with 5-aminobenzimidazole (Compound **5**) or replacing 5-aminobenzimidazole with 5-aminobenzoxazole (Compound **6**) led to inactive compounds (Table 1).

**Fig 3:**
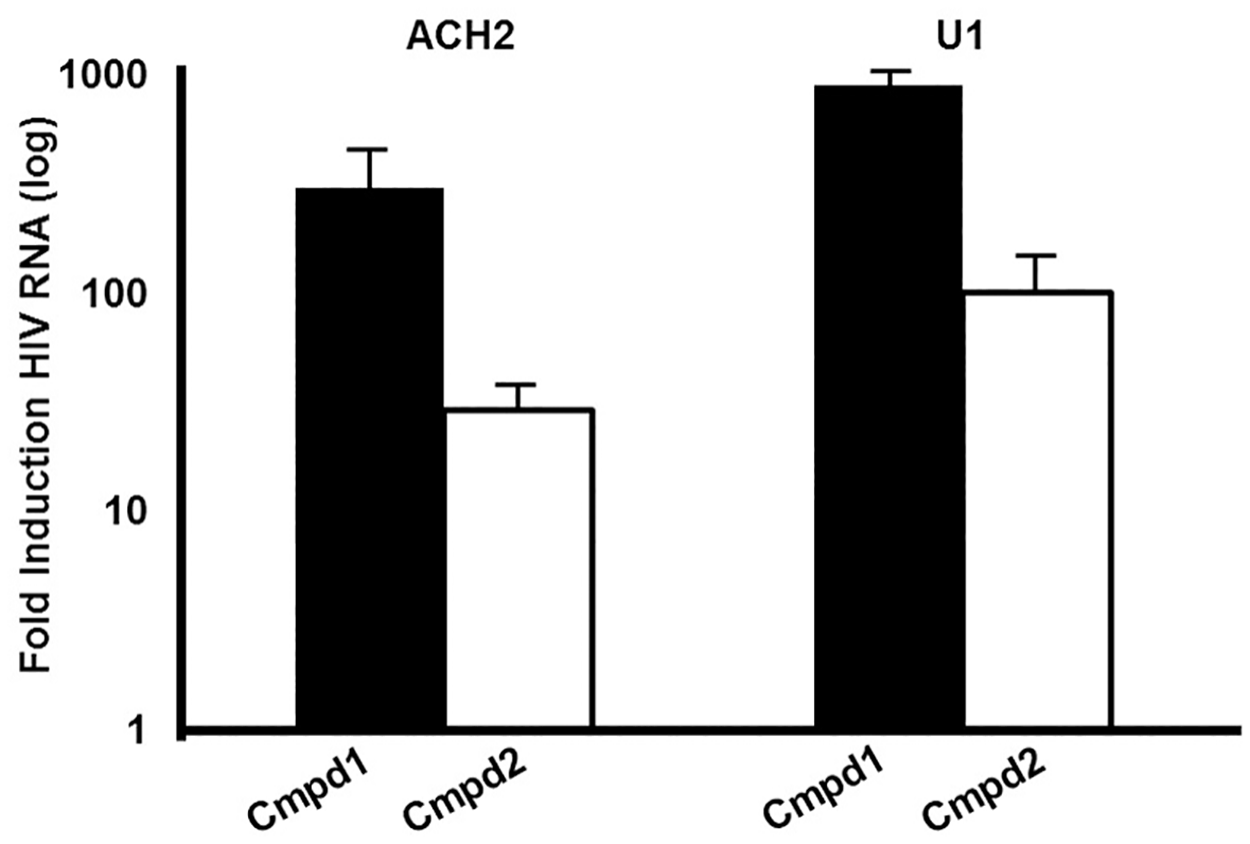
Compounds 1 and 2 induce HIV-1 transcription in ACH-2 and U1. Cells were treated with 30 μM of Cmpd 1 and Cmpd 2 for 24 h. RNA was prepared and HIV expression was monitored by qRT-PCR. Data are presented as the log fold induction over DMSO treated controls. Each bar represents treatments performed in triplicate. Error bars represent the standard error. These data are from an individual experiment that is representative of 3 independent experiments.

**Fig 4:**
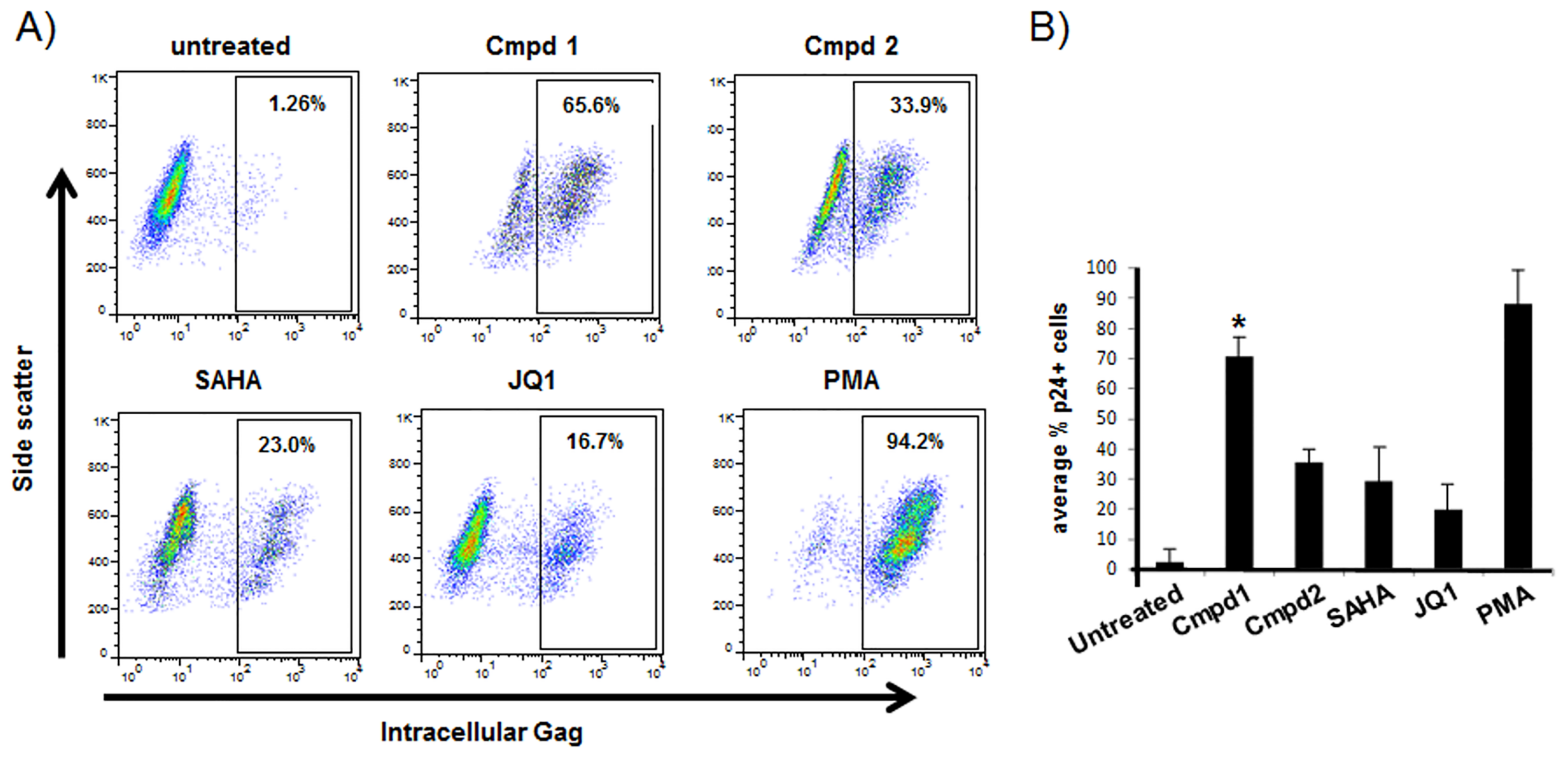
Induction of HIV-1 expressing cells following treatment with latency reversing agents. ACH-2 cells were not treated or treated with a final concentration of 30 μM Cmpd 1 or Cmpd 2, 10 μM SAHA, 1 mM JQ1 or 10 ng/ml PMA for 16 h. Cells were stained using anti-HIV-Gag-PE and analyzed by flow cytometry. Numbers within the profiles represent the percentage of positive cells. These profiles are from a single experiment. B) Data from four independent experiments. Y-axis is mean of % p24 positive cells. * Cmpd 1 p-value >0.005 compared to other treatments using a two-tailed t-test.

Compound **1** and Compound **2** also induced HIV-1 transcription in ACH-2 and U1 cell lines, two cellular models in which HIV is not efficiently expressed unless treated with cytokines or phorbol esters due to mutations in the TAR element and Tat, respectively [50,55] (Fig 3, Table 1). HIV-1 expression was induced in over 70% of ACH-2 cells when treated with Compound **1**, and Compound **1** consistently induced approximately 2-fold more cells than what was observed for reported latency reversing HDAC inhibitor SAHA or bromodomain inhibitor JQ1 [56] used at active concentrations (Fig 4). Compound **2** induced lower levels of expression of HIV-1 RNA than Compound **1** in these cell models, but induced a greater number of ACH-2 cells than did SAHA or JQ1 (Fig 4).

Cytotoxicity of test compounds was determined in SupT1 cells and stimulated PBMCs. Compounds **1** and **2** were found to have ≥ 3-fold selective windows with regards to their respective EC_2X_ (concentration for 2-fold increase in SEAP reporter), although Compound **2** proved significantly less toxic in all assays (Table 1). Fifty percent cell cytotoxicity (CC_50_) was observed for Compound **1** at concentrations of 5–18 μM whereas greater than 100 uM Compound **2** was well tolerated by T cells and PBMCs. Since they were found to be the most active compounds, Compounds **1** and **2** were selected for further characterization.

### Benzazole compounds do not activate T cells

**Fig 5:**
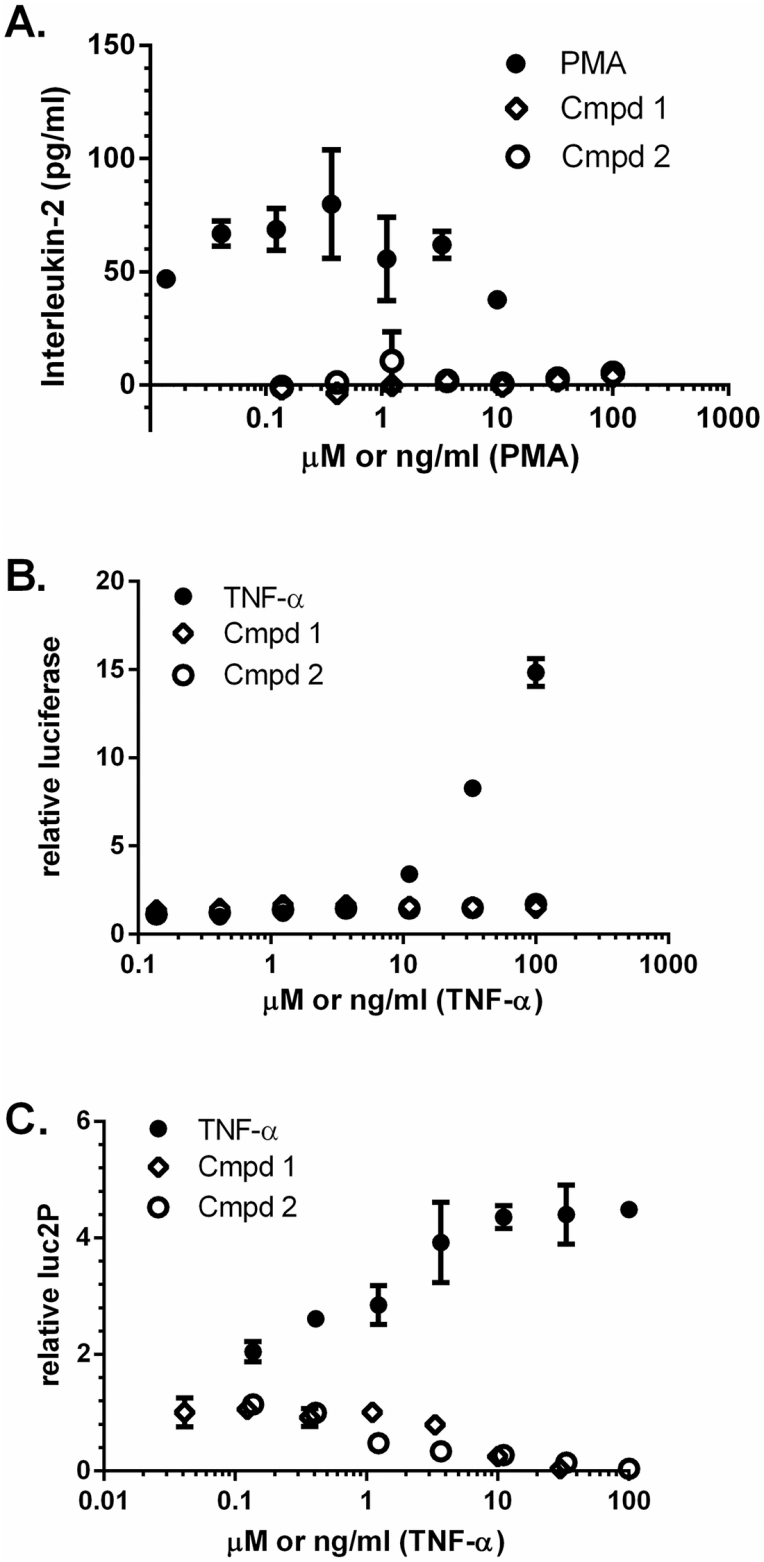
Evaluation of compounds in cytokine and transcription assays. (A) PBMCs were exposed to test compounds for 24 hours and IL-2 was quantitated by ELISA. (B) Jurkat cells expressing an NF-κB-responsive Fluc reporter were exposed to compounds and luciferase activity was measured 4 hours post addition. Results are expressed as change from vehicle-treated control. (C) HeLa cells transfected with an HIV LTR-driven Rluc reporter were treated with compounds for 24 hours. Rluc was measured and normalized to vehicle-treated control. For all panels, data is presented as the mean and standard deviation of two replicates and is representative of at least two independent experiments.

Quiescent CD4+ T cell subsets, including T memory cells, can harbor latent HIV-1, and activation of these cells with mitogens can induce proviral transcription in vitro [57–59]. To determine whether Compounds **1** and **2** activate T cells, their ability to induce interleukin (IL)-2 in treated PBMCs was examined. PBMCs were incubated with either compound for 24 h and IL-2 levels in the culture supernatant were quantified by ELISA and compared to purified IL-2 standard (Fig 5A). Whereas PMA induced high levels of IL-2, neither Compound **1** nor **2** increased secretion of IL-2, indicating that these compounds are not general T cell activators.

### Benzazole compounds do not increase expression of NF-κB- or HIV LTR-driven reporters

HIV-1 expression is highly dependent upon host transcription factors and machinery, including NF- κB [60,61]. To determine whether these benzazoles stimulate HIV-1 transcription via the NF-κB pathway, we employed a plasmid in which expression of a synthetic firefly luciferase (Fluc) reporter gene is under the control of a minimal promoter containing 5 copies of the NF-κB responsive element. This plasmid was stably transfected into Jurkat cells to generate a NF-κB human T cell reporter line. Cells were treated with TNF-α, or Compounds 1 or 2 as indicated (Fig 5B). Neither Compound **1** nor Compound **2** induced an NF-κB driven reporter in stably-transfected Jurkat cells. As expected, TNF-α strongly induced this reporter. These data suggest that benzazoles do not activate HIV-1 directly through the NF-κB pathway.

Since Compounds **1** and **2** did not induce a reporter under the control of NF-κB responsive elements, we evaluated whether benzazole compounds stimulated transcription from the HIV LTR. We constructed a plasmid in which a modified Renilla luciferase (Rluc) reporter gene is under the control of the HIV-1 LTR. This construct was transfected into HeLa cells which were then evaluated for response to test compounds 24 h post exposure. TNF-α was used as a positive control and, as expected, significantly increased expression of this reporter. Compounds **1** and **2** did not significantly increase transcription driven by the HIV-1 LTR (Fig 5C). Therefore, Compounds **1** and **2** do not increase HIV-1 transcription through stimulation of the HIV-1 LTR.

### Benzazole compounds do not act through HDAC inhibition

**Fig 6:**
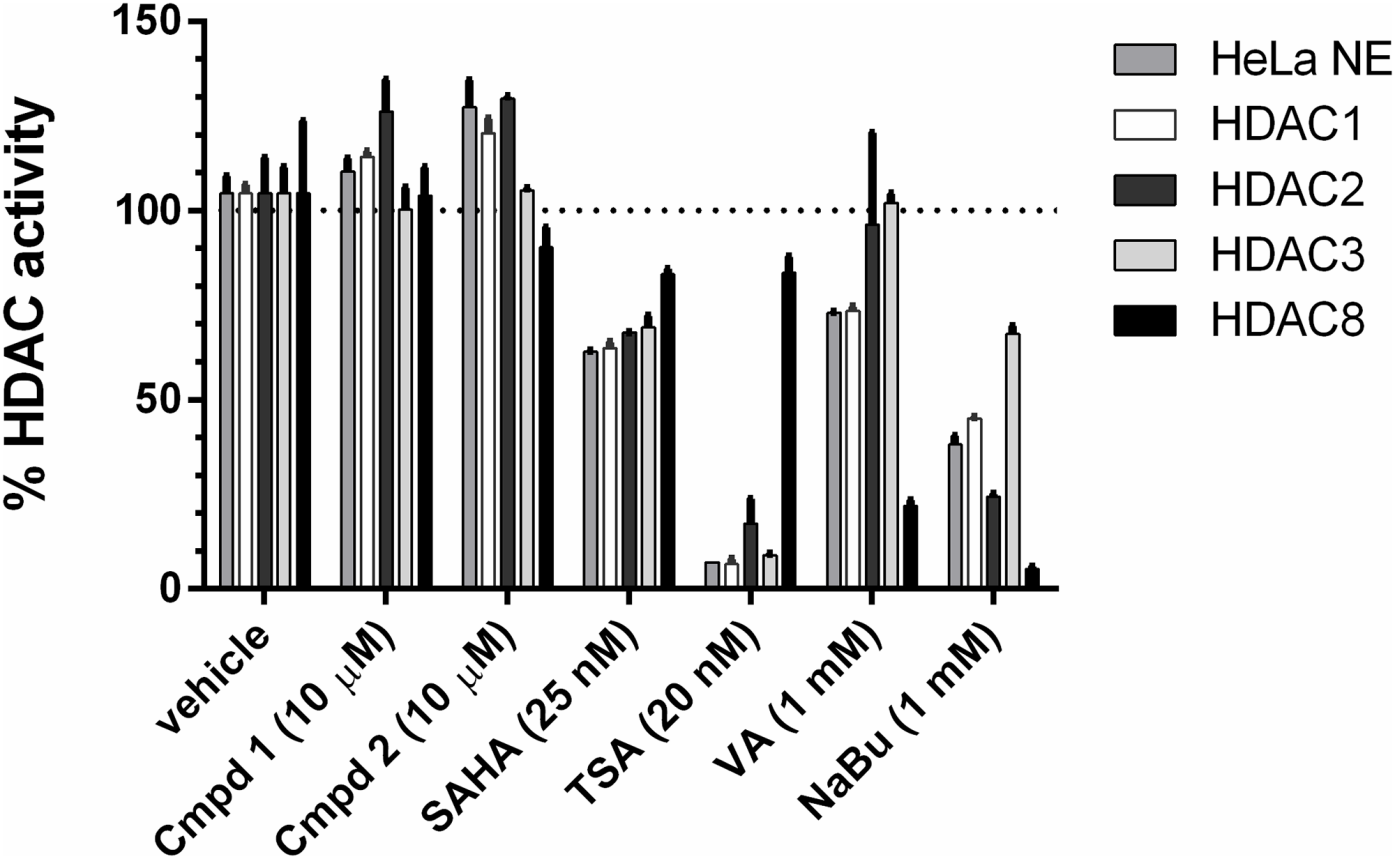
HDAC inhibition assay. Hela nuclear extract (NE) or purified HDACs were assayed for deacetylation activity in the presence of Compound **1**, Compound **2**, or known HDAC inhibitors. Data are plotted as mean ± SEM of two independent replicates and is normalized to vehicle-treated sample. Dotted line indicates 100% HDAC activity (0% inhibition). SAHA, suberanilohydroxamic acid; TSA, trichostatin A; VA, valproic acid; NaBu, sodium butyrate.

A number of HDAC inhibitors have been shown to stimulate HIV expression in cellular models of latency and some HDAC inhibitors have been evaluated clinically for eradication of latent HIV-1 [23,62]. To determine whether Compound **1** or **2** inhibit HDACs, we utilized a fluorometric based enzymatic assay in which deacetylation of a substrate generates a quantifiable fluorophore. Compounds were tested for their ability to inhibit the deacetylation activity of HeLa nuclear extracts, or of one of four purified class I HDACs [63], HDAC1, HDAC2, HDAC3, and HDAC8. A number of known HDAC inhibitors with differing inhibition profiles were included as positive controls, including SAHA, trichostatin A (TSA), valproic acid (VA), and sodium butyrate (NaBu). Compound **1** or **2** at a concentration of 10 μM demonstrated no HDAC inhibitory activity (Fig 6). This supports the conclusion that the mechanism of transcription induction by benzazoles is not through HDAC inhibition.

### Benzazole compounds do not activate latent herpes viruses

**Fig 7:**
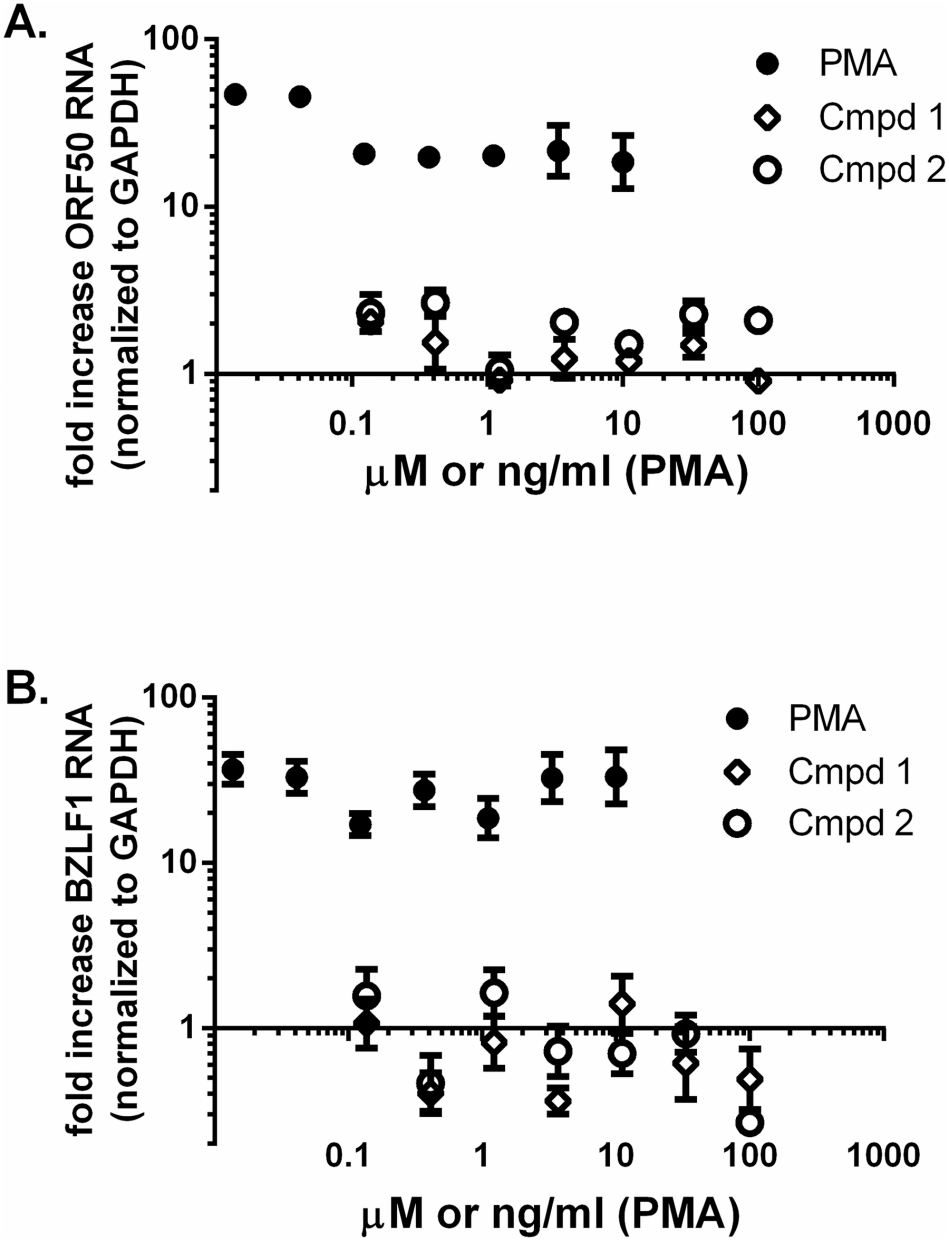
Compounds 1 and 2 do not activate latent gamma herpesviruses. BCP-1 (A) or Raji (B) cells were treated with Compound **1**, Compound **2**, or phorbol 12-myristate 13-acetate (PMA) for 24 hours and total RNA was purified. ORF50 (panel A) or BLZF1 (panel B) viral RNAs were quantitated by qRT-PCR and normalized to cellular GADPH on a per-well basis. Data are displayed as mean ± SEM fold increase compared to vehicle-treated control cells. Data point represents two independent replicates, each evaluated by qRT-PCR in duplicate.

To test the specificity of HIV-1 stimulation, we assessed the ability of these compounds to activate latent human gamma herpes viruses, specifically Kaposi's sarcoma associated herpesvirus (KSHV) and Epstein Barr virus (EBV). BCP-1 [64] and Raji [65] cells were employed as models of KSHV and EBV latency, respectively. Cells were exposed to Compound **1** or **2** for 24 h, total RNA was isolated, and viral RNA was quantified by qRT-PCR. The ORF50 (KSHV) [53] or BLZF1 (EBV) [54] latency-associated mRNAs were used as indicators of latent virus activation and were normalized on a per-well basis to human GAPDH mRNA. PMA was used as a positive control. Compound **1** or **2** at concentrations of up to 100 μM had little effect on ORF50 or BLZF1 mRNAs, while PMA stimulated both mRNAs >35-fold at concentrations as low as 0.014 ng/ml (Fig 7). These data support the conclusion that Compounds **1** and **2** are not general activators of virus expression and suggest an HIV-1 specific pathway is being targeted.

### Pharmacokinetic properties of benzazoles

The efficacy of a drug is dependent upon its in vitro potency as well as its pharmacokinetic properties and lack of off-target effects. As part of hit-to-lead profiling, compounds were assessed for pharmaceutical properties using in vitro model assays and tested for plasma exposure after oral administration to mice. These results are summarized in Tables 2 and 3.

**Table 2:** In vitro profiling properties of HIV-1 latency antagonists.

Compound	HLM/MLM^a^ stability (% loss of parent compound at 1 h)	Caco-2 P_app_ (10^−6^ cm/s)	% Plasma protein binding (human/mouse)
Compound 1	57 / 28	38	>99 / >97
Compound 2	61 / 53	4.8	>99.7 / >97.5
Verapamil	95 / 96	--	--
Disopyramide	9 / 12	--	--
Caffeine	--	28	--
Atenolol	--	0.23	--
Warfarin	--	--	99.5 / 95.3
Propanolol	--	--	74.3 / 73.8

^a^HLM, human liver microsomes; MLM, mouse liver microsomes

**Table 3:** Mouse pharmacokinetics of benzazole compounds (10 mg/kg, PO).

Compound	C_max_, plasma (μM)	AUC_0-24_, plasma (μM·h)	C_max_, brain (μM)	AUC_0-24_, brain (μM·h)
Compound 1	0.55	2.2	0.065	0.12
Compound 2	0.18	0.92	0.054	0.054

#### Bioavailability

The bioavailability of a compound in vivo is dependent on its absorption as well as metabolic stability. The Caco-2 cell line, derived from a human colon carcinoma, has characteristics that closely resemble intestinal epithelial cells and is used as a predictor of oral absorption and permeability [66,67]. Compounds with a permeability coefficient of >1 x 10^−6^ cm/sec are considered to have good oral absorption characteristics. The permeability coefficients of benzazole compounds **1** and **2** were 4.8 and 38×10^−6^ cm/s, respectively, indicating that permeability should not limit bioavailability (Table 2).

#### Metabolic stability

Enzymes such as cytochrome P450s are responsible for metabolizing drugs and are found in liver endoplasmic reticulum and microsomes [68]. To determine metabolic stability, Compounds **1** and **2** were incubated with hepatocyte preparations of human or mouse liver microsomes. Both compounds demonstrated a loss of 57–61% in human liver microsomes in 1 h supporting that this scaffold is moderately metabolically stable (Table 2).

#### Protein binding

Most small molecules bind proteins to some extent and the extent of protein binding affects the amount of free drug available for distribution into the target tissue. Therefore, a determination of the extent of binding to plasma proteins is critical for predicting the pharmacokinetic profile of a candidate drug and the doses required for efficacy. Compounds **1** and **2** were found to be highly protein bound in both mouse and human serum (Table 2), indicating that free drug concentration may be limited in vivo.

#### Pharmacokinetics

**Fig 8:**
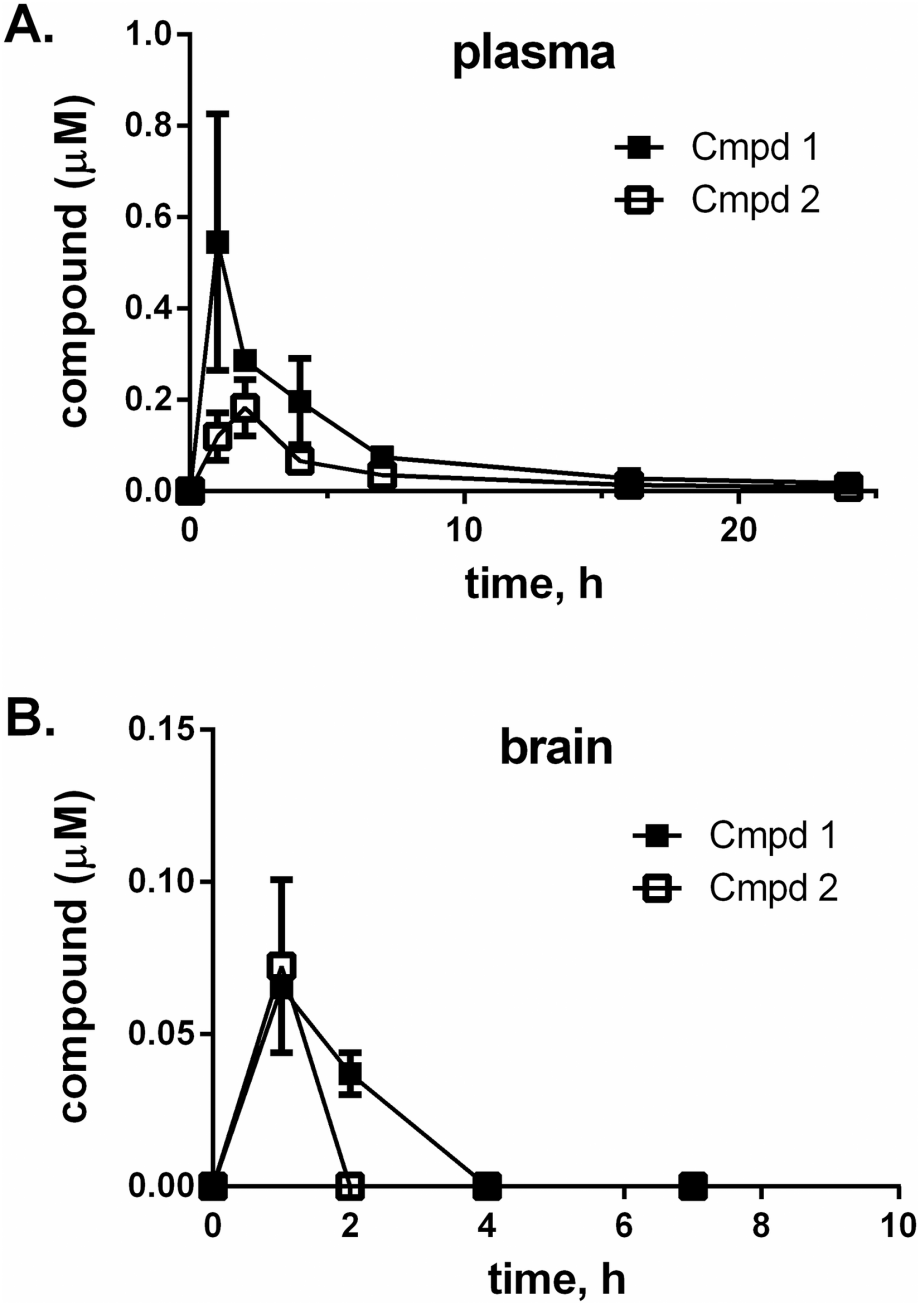
Pharmacokinetics of compounds 1 and 2 in mice. Compounds were administered as a single dose of 10 mg/kg by oral gavage. Plasma and brain were sampled at various time points for 24 hours after dosing and compound concentration was determined by LC-MS/MS. Each data point represents the mean and standard deviation of samples from three mice.

Pharmacokinetics in mice was examined after a single oral dose of 10 mg/kg. Levels of compounds were measured in the plasma and brain at time points spanning 24 h post dose (Fig 8). C_max_ in the plasma was determined to be 0.55 and 0.18 μM for Compounds **1** and **2**, respectively (Table 3) with AUC_0-24_ of 2.2 and 0.92 μM·h, respectively However, brain exposure was only a fraction of that of the plasma (Fig 8 and Table 3), suggesting that these compounds may be limited in crossing the blood-brain barrier. Taken together, these pharmacokinetics studies suggest that Compounds **1** and **2** are promising scaffolds for developing second generation benzazole compounds with improved pharmaceutical properties.

## Discussion

Efforts to eliminate latent HIV-1 in patients have met with limited success [62,69]. For example, clinical trials have demonstrated that inhibition of HDACs by administration of valproic acid, despite possibly leading to modest HIV transcription in latently infected cells, appears to have minimal impact on the viral load in vivo [41,45–47,70]. Other trials with diverse HDAC inhibitors including voriniostat [71,72], panobinostat [73], and romidepsin [74] appeared to increase transcription of HIV-1 and, in some cases, plasma viremia, but a significant reduction of the latent reservoir has not been demonstrated. Similar results were obtained in trials of the anti-alcoholism drug disulfiram [75,76].The apparent failure of these studies in reducing the frequency of latently infected CD4+ cells highlights the difficulty in targeting persistently infected cells, our poor understanding of the size and make-up of the reservoir, the inability of the therapeutic regimen to reach all latently infected cell populations, and the poor efficacy of current treatments in activating latent HIV-1. However, it is possible that more potent and specific compounds with favorable pharmacokinetics may achieve better results in patients. Recently, a small trial employing a therapeutic HIV-1 vaccine in combination with romidepsin reported a decrease in the HIV-1 reservoir in patients measured by viral outgrowth assay [77].

Another confounding factor is that the maintenance of HIV-1 latency may depend upon multiple processes. RNA polymerase (RNAP) processiveness, chromatin organization, transcriptional interference, tissue specific transcriptional regulators, and local DNA structure at the integration site are just a subset of mechanisms that have been implicated in limiting HIV-1 transcription and maintaining latency. It is possible that a combination of latency antagonists that act via different mechanisms will be necessary to completely reactivate all latent reservoirs in the patient [34,78]. Combination approaches have been shown to enhance induction of HIV expression in various latency models [79–82]. Furthermore, minimally effective small molecules may synergistically increase the activity of known latency reversing agents [83].

Since a combination of therapeutics with different mechanisms of action may be necessary to target different reservoirs of latently infected cells in various tissue compartments, we set out to identify compounds capable of specifically activating HIV-1 transcription. We have identified a novel class of benzazole compounds that activate HIV-1 proviral transcription in cellular models of latency. We present data that these compounds induce HIV transcription in several cell types and initial pharmacokinetic studies suggest that they may provide a scaffold for further optimization.

The mechanism of action for these compounds appears to be unique compared to that of other latency reversing agents including HDAC inhibitors, such as SAHA (vorinostat), valproic acid, and the hydroxamic acid, panobinostat; BET bromodomain inhibitors including JQ1; and protein kinase C modulators such as prostratin. Taking our data together, the identified compounds do not act as general activators of T cells and induce only modest changes to NF-kB signaling. Importantly, these compounds do not inhibit HDAC activity and do not increase LTR-dependent transcription. Since Compounds **1** and **2** are as active as those compounds that target transcriptional mechanisms of latency and appear to act through a unique pathway(s), they may hold potential to complement current latency reversing agents. Importantly, these compounds did not induce cellular latency models of gamma herpes viruses, demonstrating selectivity to HIV-1. Although further investigation is necessary to determine the precise mechanism of action of these compounds, a small molecule could potentially act through a number of pathways such as alteration of chromatin structure, interaction with key transcription factors, inhibition of repressors such as NELF, or increased RNA processivity.

We have presented discovery and early development efforts of latency reversing compounds. Through pharmacokinetic profiling, we have identified potential concerns with protein binding, metabolic stability, and plasma exposure after oral dosing. Additionally, initial exploration of the structure-activity relationship demonstrated limited tolerance for changes to the position of the benzoxazole amine substituent. Further optimization of this scaffold with the goals of limiting metabolism and reducing protein binding may improve pharmacokinetic properties and allow for future proof-of-concept studies in an animal model of HIV latency.
